# RET Receptor Tyrosine Kinase: Role in Neurodegeneration, Obesity, and Cancer

**DOI:** 10.3390/ijms21197108

**Published:** 2020-09-26

**Authors:** Arun Kumar Mahato, Yulia A. Sidorova

**Affiliations:** Institute of Biotechnology, University of Helsinki, HiLIFE, Viikinkaari 5D, 00014 Helsinki, Finland; arun.mahato@helsinki.fi

**Keywords:** rearranged in transfection (RET), neurodegeneration, obesity, cancer, RET agonist, neurorestoration, retinitis pigmentosa, hirschsprung disease, glial cell line-derived neurotrophic factor (GDNF) family ligands (GFLs), growth differentiation factor 15 (GDF15)

## Abstract

Rearranged during transfection (RET) is the tyrosine kinase receptor that under normal circumstances interacts with ligand at the cell surface and mediates various essential roles in a variety of cellular processes such as proliferation, differentiation, survival, migration, and metabolism. RET plays a pivotal role in the development of both peripheral and central nervous systems. RET is expressed from early stages of embryogenesis and remains expressed throughout all life stages. Mutations either activating or inhibiting RET result in several aggressive diseases, namely cancer and Hirschsprung disease. However, the physiological ligand-dependent activation of RET receptor is important for the survival and maintenance of several neuronal populations, appetite, and weight gain control, thus providing an opportunity for the development of disease-modifying therapeutics against neurodegeneration and obesity. In this review, we describe the structure of RET, its signaling, and its role in both normal conditions as well as in several disorders. We highlight the differences in the signaling and outcomes of constitutive and ligand-induced RET activation. Finally, we review the data on recently developed small molecular weight RET agonists and their potential for the treatment of various diseases.

## 1. Introduction

Receptor tyrosine kinases (RTKs) are transmembrane proteins conveying extracellular stimulus inside the cell. The members of RTKs are expressed in almost every if not all cells in the organism and play pivotal roles in different cellular functions such as proliferation, cellular differentiation, cell survival, cell migration, and metabolism. There are 58 different RTKs in humans with similar molecular structure, which are activated by ligands binding to their extracellular domain [1].

All RTKs have an extracellular domain that interacts with a ligand (directly or indirectly) and an intracellular kinase domain that is activated upon ligand binding and catalyzes autophosphorylation. These two domains are connected by a transmembrane domain. Apart from these three structural domains, there is a juxtamembrane domain that was initially thought to be just a mechanical linker between two parts of the protein. However, recent studies show that it may also regulate the function of at least some RTKs [2].

Ligand interaction with extracellular domains of RTKs promotes their dimerization or oligomerization and triggers the phosphorylation of tyrosine residues in their kinase domains. Phosphorylated tyrosine residues recruit adapter proteins and trigger the activation of intracellular signaling cascades [1].

The main objective of this review is to highlight the importance of rearranged in transfection (RET) in health and disease conditions. The present review is focused on the structure, function, and role of RET in neurodegeneration, obesity, and cancer. Furthermore, this review updates recent findings on how RET can be targeted with small molecules for the treatment of various disease conditions. RET is unique as unlike other RTKs, it does not bind to the ligand directly. Instead, it forms a tripartite complex consisting of a dimeric ligand, two molecules of ligand-binding co-receptors (either glial cell line-derived neurotrophic factor (GDNF) family receptor alpha (GFRɑ) or GDNF family receptor alpha-like (GFRAL) and two molecules of RET. This, on one hand, provides an opportunity to target it selectively in diseases via co-receptors or the interaction surface between a co-receptor and RET. This is important because the kinase domain of RET is structurally similar to the kinase domains of other RTKs; therefore, it is difficult to find selective molecules acting via the kinase domain. On the other hand, GFRɑ1 can regulate RET signaling in a way that for a particular stimulus, signaling bias may exist [3], and this may allow RET to orchestrate cellular processes more precisely. However, disturbances in cellular levels of RET and GFRɑ co-receptors can lead to undesirable consequences, such as RET activation in the absence of a ligand, which can potentially result in the formation of malignant tumors [4,5].

## 2. RET Receptor Tyrosine Kinase

**Figure 1:**
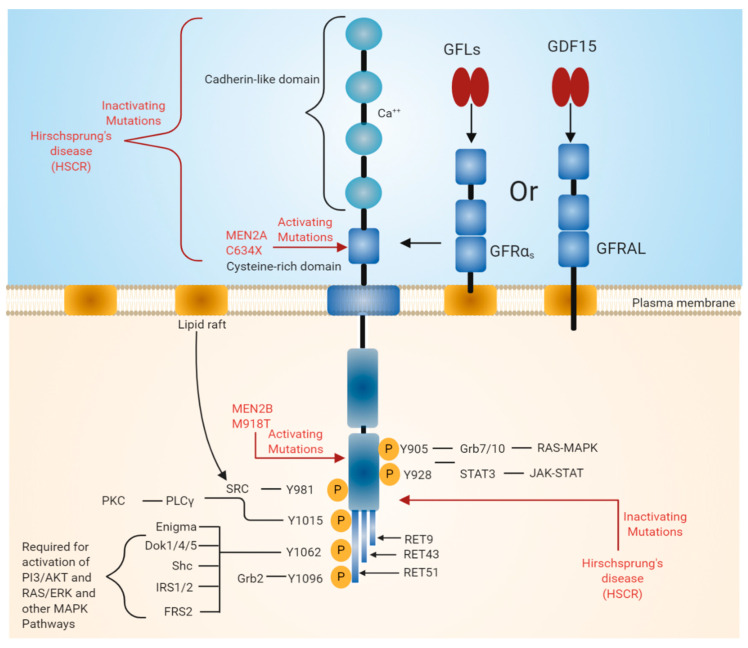
Rearranged in transfection (RET) receptor structure and its intracellular signaling pathway. The extracellular domain of RET contains four cadherin-like repeats and a cysteine-rich domain. Ca^2+^ ions bind to the extracellular cadherin-like domains of RET, which is required for its activation. The intracellular domain of RET contains a typical kinase domain. RET has three isoforms (RET9, RET43, and RET51), which differ in their carboxy-terminal amino acids. RET9 and RET51 are evolutionarily highly conserved. RET is phosphorylated at multiple tyrosine residues when activated by different ligands. Phosphorylated tyrosine residues serve as docking sites for various adaptor proteins that induce the activation of downstream signaling pathways essential for cell growth, proliferation, survival, differentiation, or appetite control. The black line indicates the binding of adapter protein and the activation of downstream signaling pathways. The red line indicates mutations in the RET region that are responsible for diseases such as multiple endocrine neoplasia (MEN) syndromes 2A and 2B and Hirschsprung disease (HSCR).

RET was identified as an oncogene activated by the recombination of DNA [6,7]. Unlike other RTKs, RET contains cadherin-like repeats in the extracellular domain (Figure 1). The N-terminal region of RET consists of four cadherin-like domains (CLDs 1-4) each of 110 amino acid residues and a cysteine-rich region. The calcium-binding site is present between CLD2 and CLD3. The N-terminal region of RET encodes a signal sequence (1–28 residues) that directs RET to the endoplasmic reticulum (ER). The extracellular domain of RET possesses 12 glycosylation sites that undergo extensive glycosylation in ER to form 150 KDa RET. The further modification of RET occurs in Golgi to form 170 KDa mature RET. Glycosylation increases the stability of the mature RET [8,9]. Inactivation mutations in the intracellular and extracellular domains are associated with Hirschsprung’s disease (HSCR disease), which is explained later in the text (see Section 3.3). The binding of ligand is calcium dependent, and calcium ions are required for the RET–ligand complex formation, which further induces RET autophosphorylation [10,11]. Furthermore, calcium is necessary for the proper folding of RET in the ER [12].

The extracellular region also includes 120 residues of the cysteine-rich region, which is adjacent to the transmembrane domain. The intracellular domain of RET contains a typical kinase domain. The alternative splicing of RET results in three different protein isoforms, i.e., RET9 (1072 amino acids), RET43 (1106 amino acids), and RET51 (1114 amino acids) [13,14]. In most tissues, all these isoforms are co-expressed. However, the expression of the RET9 isoform is much higher than that of the RET51 isoform, while the expression of RET43 is much lower compared to the RET51 isoform [14]. The targeted mutagenesis of the mouse genome, which either expresses RET9 or RET51, revealed that mice lacking RET51 are viable and appear normal, whereas mice lacking RET9 have defects in the innervation of the gut and renal development [14,15]. The ligand-induced dimerization of RET leads to the autophosphorylation of various tyrosine residues and further activates intracellular signaling cascades, which affects a number of cellular processes [3].

In normal conditions, RET is activated by glial cell line-derived neurotrophic factor (GDNF) family ligands (GFLs). GFLs belong to the transforming growth factor beta (TGFb) superfamily. Traditionally, four proteins—GDNF, neurturin (NRTN), artemin (ARTN), and persephin (PSPN)—were referred to as GFLs. Recently, another protein, GDF15, was shown to signal via RET. GDF15 is a distant member of the TGFb superfamily with a close relationship with GFLs. Similar to other TGFb members, GDF15 also includes a highly conserved pattern of seven cysteine residues in its mature domain. Of the seven cysteine residues, six form highly stable intra-chain disulfide bonds, and the remaining one forms an inter-chain disulfide bond. Similar to other members, GDF15 is secreted as a dimeric protein. Therefore, it can be considered as a 5th GFL [16,17].

GDNF at first binds glial cell line-derived neurotrophic factor family receptor alpha 1 (GFRɑ1) and consequently forms a tripartite complex with RET. Other members of GFLs such as NRTN binds to GFRɑ2, ARTN binds to GFRɑ3, and PSPN binds to GFRɑ4 in order to form a complex with RET and induce signaling [18,19]. However, preferences of the ligand and co-receptor might change, making the co-receptor unselective. For example, GDNF can also bind to GFRɑ2 and NRTN, ARTN, and PSPN can also bind to GFRɑ1 [20,21,22]. GDF15 binds to a distant orphan member of the GFRɑ family called GFRAL and further forms a complex with RET. Interactions between GDF15 and GFRɑ co-receptor are not reported [23,24,25,26].

The binding of GFLs to GFRɑ co-receptors recruits two molecules of RET receptor into lipid rafts [27,28]. As a result, the formation of a signaling complex is completed and the trans autophosphorylation of tyrosine residues on the intracellular domain of RET occurs. The intracellular domain of RET contains 12 autophosphorylation sites: Y687, Y752, Y806, Y809, Y826, Y900, Y905, Y928, Y981, Y1015, and Y1062 (Figure 1). The phosphorylated tyrosine residues serve as a docking site for several adapter proteins, which in turn activate internal signaling. Y905 is the docking site for Grb7/10. Y1096, which is unique and present only in RET51 long isoform, is the docking site for Grb2, Y1015 is the docking site for phospholipase C, and Y981 is the docking site for c-Src. Y1062, which is present at the carboxy terminal of RET, serves as a docking site for several adapter proteins such as Shc, insulin receptor substrate 1/2 (IRS1/2), fibroblast growth factor receptor substrate 2 (FRS2), downstream of tyrosine kinase ⅘ (DOK1/4/5), and Enigma [18,29]. The phosphorylation of Y1062 activates multiple downstream signaling pathways such as mitogen-activated protein kinase (MAPK) pathways RAS/extracellular signal-regulated kinase (ERK), p38MAPK, phosphatidylinositol-3-kinase (PI-3K)/AKT, and Rac/c-Jun N-terminal kinase (JNK) pathways. The activation of these downstream signaling pathways is necessary for cell survival, differentiation, proliferation, motility, and functioning [16,18,30].

## 3. Role of RET in Various Disease States

**Figure 2:**
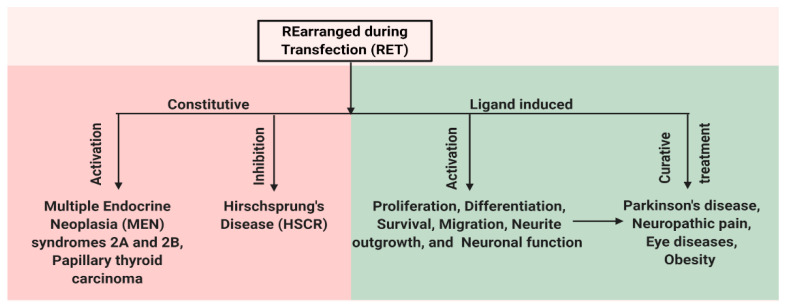
Schematic representation of multiple facets of RET protein. RET has a role in both disease and normal state. Constitutive activation of RET by mutations leads to human multiple endocrine neoplasia (MEN) syndromes 2A and 2B, while mutations that inhibit RET activation can cause Hirschsprung disease (HSCR). Ligand-based activation of RET is essential for the development of both peripheral and central nervous systems and also outside the nervous system. Therefore, targeting RET with agonists can be a useful approach in the treatment of neurodegenerative diseases and obesity, and RET antagonists may have a role in the therapy of RET-dependent cancers.

RET has an important role for the normal development of both peripheral and central nervous systems and also has functions outside the nervous system. In the central nervous system, RET is expressed in the ventral midbrain, ganglia layer of retina and olfactory epithelium, undifferentiated neuroepithelial cells of the ventral neural tube, spinal cord, and the hindbrain [31,32,33]. In the adult brain, the expression of RET is restricted to the midbrain, cerebellum, pons, and thalamus [34]. The expression of RET is also observed in the kidney, thyroid, and lungs [35]. Mutations in RET change the activity of the receptor and result in various diseases. Mutations that lead to the constitutive activation of RET result in human multiple endocrine neoplasia (MEN) syndromes 2A and 2B, while mutation that inhibits RET activation can cause Hirschsprung disease (HSCR) [5,36]. In addition, the ligand-dependent activation of RET can be important for treating various disease conditions caused by neuronal degeneration or the disturbances of functional activity of neurons, e.g., Parkinson’s disease (PD), neuropathic pain, retinitis pigmentosa (RP), and obesity (Figure 2). Here, we present a detailed review on the role of RET in various disease states.

### 3.1. Normal Function of RET in DA Neurons and Its Implication in Parkinson’s Disease

The physiological role of RET has been extensively studied in dopamine neurons because GDNF was discovered as a survival factor for these cells [37]. Later, RET was identified as the receptor of GDNF through which it triggers the neurite outgrowth and the survival of the central nervous system neurons [38]. In mice, RET is expressed in ventral midbrain dopamine neurons from 12.5 days postcoitum (dpc) until birth and it remains expressed throughout the lifespan [31].

Constitutive Ret knockout mice die shortly after birth due to the absence of kidney. However, these mice have normal midbrain dopamine neurons [39,40], suggesting RET as a dispensable receptor for the embryonic development of dopamine system. Since RET is the major receptor for GDNF signaling, the function of RET was studied in adult midbrain dopamine neurons by the selective ablation of RET genes. Among two different studies conducted, both groups reported no change in the survival of dopamine neurons during the first 9 months of mouse life [41,42]. However, Kramer et al. reported the progressive and late degeneration of dopamine neurons in RET conditional knockout mice compared to the age-matched controls when experimental animals were monitored for a period of two years. Further, they reported that a loss of neurons was accompanied by inflammation and gliosis. These data delineate RET as an important regulator for long-term maintenance of the nigrostriatal adult dopamine system [42].

MEN2B, an inherited cancer syndrome that is described in more detail in Section 3.6, is often caused by the presence of constitutively active RET as a result of point mutation in the gene encoding this RTK. In mice overexpressing a variant of the RET gene with a mutation causing MEN2B, the levels of dopamine and dopamine metabolites were found to be increased in different brain regions, including the striatum. In addition, the level of tyrosine hydroxylase (TH, a key enzyme of dopamine synthesis) protein, and TH mRNA levels were also increased along with the number of TH-positive cells in the substantia nigra pars compacta (SNpc), suggesting the importance of RET activity in maintenance of the dopamine system [35,43].

We have also shown that RET is required for the survival of naive cultured dopamine neurons as well as for neuroprotection when challenged with neurotoxin. Both RET agonist BT13 and GDNF do not promote the survival of cultured embryonic dopamine neurons lacking RET. Furthermore, both BT13 and GDNF protect dopamine neurons from 6-OHDA and MPP+ neurotoxin-induced cell death only when they express RET [44,45]. Recently, RET signaling activated by its ligand GDNF has been shown to prevent Lewy pathology in midbrain dopamine neurons, which further highlights the importance of RET for the maintenance of dopamine systems [46].

PD is a progressive neurodegenerative disease that affects most profoundly the dopamine neurons in the substantia nigra pars compacta (SNpc) [47]. The loss of dopamine neurons results in a deficiency of dopamine, which then induces motor impairment. Motor disturbances serve as diagnostic symptoms of PD. Other neuronal populations all over the body are also affected, and their loss or dysfunction cause non-motor symptoms that can precede motor symptoms by several years and even decades. There are no drugs to cure PD. Current therapy provides only symptomatic treatment to the PD patients.

Due to the importance of RET signaling in the dopamine system as highlighted above, GFLs have been tested both in preclinical and clinical settings. GFLs were found to promote the survival of midbrain dopamine neurons both in vitro and in vivo [37,48,49]. Furthermore, GFLs provide both neuroprotection and neurorestoration when studied in various toxin-based models of PD in rodents and primates [48,50,51,52,53,54,55,56,57]. Based on the promising results in preclinical studies, clinical trials were conducted with GDNF and NRTN. However, the outcomes of the clinical trials performed with GFLs in PD patients are inconclusive. PhaseI/II clinical trials conducted using recombinant GDNF and adeno-associated virus 2 encoded NRTN (AAv2-NRTN, CERE-120) indicated that both treatments were well-tolerated. The improvement in motor performance of at least some patients was seen along with an increase in [18F] DOPA uptake in the brain [58,59,60]. The last parameter indicates an increase in function and likely the level of dopamine transporter, which suggests the restoration of dopamine neuron terminals into putamen.

Despite promising preliminary data, in double-blinded placebo-controlled trials with both of these GFLs, a statistically significant improvement in motor function of patients was not achieved. However, an increase in [18F] DOPA uptake in the brains of PD patients was detected [61,62,63,64]. According to the data from the double-blinded placebo-controlled study carried out with AAv2-NRTN, early-stage PD patients benefited from the treatment more when compared to advanced-stage PD patients [65]. Moreover, post hoc analysis of recent clinical trials with GDNF revealed an improvement in motor function in 43% of the patients treated with GDNF [63,64]. We have provided a detailed review of the results of clinical trials conducted with GDNF and CERE-120 in our previous review [66].

While GFLs proteins had limited success in clinical trials in PD patients, targeting RET can still be a valid approach for PD treatment. The poor tissue distribution of GFLs caused by their binding to heparan sulfate proteoglycans might have resulted in partial coverage of the putamen in PD patients, which was insufficient to observe statistically significant improvement in motor scores. The main participants of clinical trials with GFLs were late-stage PD patients. As a result of ethical reasons associated with the invasiveness of GFL delivery, it is very difficult to recruit early-stage patients into these clinical trials. In the brains of these patients, most of the dopamine cell bodies and fibers have already degenerated, and hence they are unlikely to benefit from GFL-based therapy [53]. The problems associated with GFLs delivery into the brains of PD patients can be solved by developing small molecule RET agonists with better pharmacokinetics and pharmacodynamics properties crossing the blood–brain barrier. This will allow including early-stage PD patients into clinical trials. Thus, targeting RET in PD patients can be a disease-modifying strategy, but further research is needed to reach this goal.

### 3.2. Retinitis Pigmentosa and Other Eye Diseases

RP is a rare genetic disorder with a prevalence of approximately 1:4000, which is caused by the degeneration of photoreceptors in retina [67,68]. Degeneration starts from rods on the periphery, but at the latter stage, cones in the macula and fovea are also affected. Symptoms include loss of night and peripheral vision, which worsen with time, leading eventually to complete blindness. The death of photoreceptors is accompanied by the accumulation of pigment on the periphery of retina seen during ophthalmological examination [67,69]. The condition is incurable. Some reports suggest protective effects of vitamin A and fish oils in RP patients, but the recent Cochrane systematic review concludes that the benefits of these treatments are uncertain [68]. The genetics of RP is diverse and complex; mutations in more than 40 genes were found to be associated with the disease [70]. This complicates the development of gene-therapy based approaches to treat RP.

In some animal models of RP, AAv-encoded GDNF slowed down the morphological and functional deterioration of retina [71,72]; however, high levels of GDNF secretion accelerated the degeneration of photoreceptors. Other authors failed to see protective effects of GDNF in animal models of RP [73,74], while detecting an effect of RET activation by e.g., small molecular weight agonist [73]. A lack of GDNF efficacy in RP can be related to the level of transgene expression [73] or poor diffusion of the protein in the eye [74]. GDNF also exerted trophic effects toward axotomized retinal ganglion cells [75], thus unraveling its potential usefulness for the treatment of glaucoma. These results establish RET as a target for novel therapeutics in above-mentioned eye diseases.

GDNF-supportive effects toward photoreceptors are indirect and mediated by retinal glial cells (Müller cells), which express both GFRɑ and RET [30,31]. Müller cells secrete various trophic factors and are critical for the survival of retinal neurons in diabetes. Therefore, targeting GDNF receptor RET with either protein, peptide, or small molecule ligand potentially can also slow down the progression of diabetic retinopathy [76,77].

### 3.3. Hirschsprung Disease (HSCR)

HSCR is a rare disease (population incidence 1:5000 live births) caused by a disturbance in the development of the enteric nervous system and characterized by the absence of enteric ganglion cells in a part of the lower gastrointestinal tract that is variable in length [36,78]. The main treatment strategy is surgical removal of the affected portion of intestine, but the motility problems remain, thus limiting the long-term therapeutic efficacy of this approach.

Genetic factors play a major role in the pathogenesis of HSCR, with RET being the primary gene associated with the disease. Mutations in RET were found in approximately 50% of patients with familial HSCR and up to 20% of sporadic cases [78]. According to recent metaanalysis data, mutations associated with HSCR can occur almost in any site of Ret, but they are most commonly found in exons 13 (11.32%), 15 (7.55%) (both coding RET kinase domain), and 10 (7.55%) (coding a part of cystein-rich domain) [79,80]. These mutations are inactivating; they abrogate RET signaling, which leads to the prevention of neural crest cell migration and distortions of the enteric nervous system. The earlier in development mutation occurs and neural crest cell migration is blocked, the longer aganglionic segment will be [80]. In animal models, the down-regulation of GFRɑ1 expression also resulted in HSCR [81] and in biopsies of a subset of HSCR patientsa reduced level of GFRɑ1 protein was detected, further supporting the role of the GFL/GFRɑ/RET axis in the development of this condition [82].

### 3.4. Neuropathic Pain

Neuropathic pain is defined by the International Association for the Study of Pain as a “pain that arises as a direct consequence of a lesion or diseases affecting the somatosensory system” [83]. It affects up to 10% of adults [84] and imposes significant economic burden on the society. According to Schaefer et al., the estimated total annual costs of neuropathic pain were equal to 27,259 USD per patient [85]. Neuropathic pain can appear as a result of traumatic nerve lesion, disease e.g., diabetes, viral infection, cancer or as a side effect or treatment with e.g., anticancer drugs or opioids [86,87], and it is more common in women and the elderly [86]. Thus, due to an increase in the prevalence of underlying conditions and aging population, the number of affected people is expected to grow in the future.

The treatment of neuropathic pain is a challenge for healthcare professionals. Available drugs poorly manage the condition. Any given analgesic produces at least 50% pain in less than 30% of patients [88] and with any combination of existing drugs, adequate pain control can be achieved in approximately half of patients. Tolerance and dependence are common side effects of currently available analgesics. Neither of the drugs used nowadays to treat neuropathic pain is considered disease-modifying.

In neuropathic pain states, sensory neurons are damaged. RET and GFRɑ co-receptors are expressed in a significant portion of healthy sensory neurons, and their expression is upregulated after lesion in rodents [89]. Up to 80% of human sensory neurons express RET [90]. GFLs promote the survival of sensory neurons and therefore have a disease-modifying potential in neuropathic pain. However, their involvement in nociception is complex, and effects can depend on the dose, administration schedule, administration site, condition of animals, and a disease model. In our recent review, we described these issues in detail [66].

In several models of neuropathic pain, GDNF and ARTN were shown to provide analgesic effect and restore lesioned sensory neurons [91,92,93], thus showing disease-modifying potential. However, in inflammatory models, they seem to increase pain. In recent clinical trials, good tolerability and the efficacy of ARTN in neuropathic pain patients was observed. However, the dose–response curve was biphasic [94]. Importantly, ARTN provided pain relief in a population of patients resistant to the therapy with at least two standard analgesics [94]. These patients are difficult to treat and truly in need of novel drug classes.

Adverse events seen in clinical trials mainly included changes in temperature perception, headache, pruritus, and rash, and they were mild or moderate in severity [95]. Since GFLs can signal also through different receptors than RET, some effects of GFLs in the sensory system, e.g., cold-induced pain, can be non-RET mediated [96,97].

A single cell transcriptome analysis of mouse sensory neurons revealed 11 subtypes of these cells. RET was expressed in low-threshold mechanoreceptors responsible for pain elicited by mechanical stimulation (which is often tested in preclinical models), neurons responsive for itchy feeling, and some others [98]. Thus, pruritus reported in some patients treated with ARTN in clinical trials as an adverse event is likely RET mediated.

It is clear that RET- and GFL- signaling plays an important role in pain and analgesia. However, more data are needed to understand the exact action of each component of the GFL/GFR/RET axis in these processes. The results of clinical trials are promising. Research focused on understanding the molecular and cellular consequences of RET activation in the sensory system, as well as on the evaluation of efficacy and safety of RET targeting molecules in preclinical and clinical settings, is important for the development of novel disease-modifying treatments against neuropathic pain.

### 3.5. Role of RET in the Non-Homeostatic Regulation of Body Weight

Obesity and overweight are the conditions defined as excessive fat deposition that can result in diabetes, cardiovascular diseases, osteoarthritis, and cancer. According to the World Health Organization (WHO), in 2016, 1.9 billion people were overweight, and among them, 600 million people were obese. Body weight and feeding behavior are regulated both by homeostatic and non-homeostatic control mechanisms. Under homeostatic conditions, feeding behavior and energy metabolism are controlled by hypothalamic neural circuits by integrating nutrient and hormonal signals from the periphery [99]. However, during stress conditions, an organism uses an alternative program in order to achieve metabolic changes [100]. Recently, GDNF receptor alpha-like (GFRAL), which is expressed in the neurons of the area postrema and nucleus of the solitary tract, has been identified as the target receptor for GDF15 that regulates food intake during stress conditions. GFRAL-GDF15 requires RET as a signaling receptor through which it regulates body weight [23,24,26]. Intriguingly, RET is expressed in the area postrema and nucleus of the solitary tract of rodents and human [101]. However, RET phosphorylation and its downstream signaling events in GFRAL-positive neurons remain to be elucidated.

### 3.6. Role of RET in Cancer

RET was discovered as a protooncogene, and its oncogenic potential has always been acknowledged. A lot of research has been conducted on mutated constitutively active forms of RET, which play a major role in thyroid cancer, pheochromocytoma, and parathyroid hyperplasia, as well as in the development of lung cancer in a subset of patients. In recent years, the reports regarding the role of wild-type RET activated by its cognate ligands in the progression of tumors originating from other tissues started to appear [102,103], but this field is much less studied, and final conclusions are yet to be made.

Clinical features of RET-dependent cancers and extensive data on RET expression in different tumor types are reviewed elsewhere [29,102,103,104]. In the present review, we focus rather on neglected aspects of GFL/GFRɑ/RET signaling in the context of oncogenic transformation providing only a minimal background on the above-mentioned issues. In particular, we discuss the differences between the constitutive and ligand-induced activation of RET and possible involvement of GFL co-receptors in tumor progression and invasion.

#### 3.6.1. Oncogenic Potential of Constitutively Active Oncogenic Forms of RET

The ligand-independent activation of RET is caused by gain-of-function mutations manifesting clinically as MEN2 or the formation of a fusion protein containing the intracellular kinase domain of RET and N-terminal domain from another protein with the ability to dimerize, resulting in the development of papillary thyroid carcinoma (PTC) [102,105]. RET bearing gain-of-function mutations is constitutively active and continuously stimulates signaling cascades such as ERK and PI3K/Akt in the cells promoting proliferation, survival, and metastasis [106]. In contrast to physiological conditions, the activation mechanisms in the above-listed intracellular cascades in the presence of mutated RET are not balanced by the negative regulation mechanisms, further contributing to the process of oncogenic transformation of the cell [107].

MEN2 is diagnosed in 5–10% of thyroid cancer patients and includes three conditions: MEN2A accounting for the vast majority of MEN2 cases, familial medullary thyroid carcinoma (FMTC) occurring in 10–20% of MEN2 patients, and MEN2B identified in approximately 5% of MEN2 patients. All MEN2 patients have medullary thyroid carcinoma, and approximately 50% of MEN2A and MEN2B patients also develop pheochromocytoma. In addition, 20–30% of MEN2A patients also have parathyroid disease. The MEN2B phenotype is the most aggressive, has early onset, and if untreated by thyroidectomy, it leads to death in half of patients by the age of 25 years. Patients with MEN2B mutation in RET (mainly Met918Thr) are recommended to undergo prophylactic thyroid surgery during their first year of life, while for others, surgery is suggested within the first 5 years of life or even later [104]. Mutations in RET are identified in approximately 50% of patients with sporadic medullary thyroid carcinoma [25].

In patients with MEN2A, mutations occur in the extracellular domain of RET and lead to the ligand-independent formation of a covalent dimer, whereas in patients with MEN2B, the mutations typically occur in the RET kinase domain and are accompanied by an activation of monomeric form. The most common mutation leading to MEN2A is C634X, and the most common mutation leading to MEN2B is M918T [103]. In FMTC, mutations are found in both intracellular and extracellular domains of RET [103,105].

PTC is the most common thyroid cancer. It is associated with RET rearrangements in 35% of patients from North America, and in other populations, it can vary from 25 to 65% [103,108]. A higher incidence of RET/PTC rearrangement is seen in children, and upon exposure to radioactive iodine isotopes [109], for instance, RET/PTC rearrangements were identified in 51.3–77% of tumor specimens collected from 5–18-year-old children exposed to radiation after Chernobyl reactor meltdown, while in non-exposed children, their prevalence was below 40% [109].

RET kinase domain fusion with kinesin family member 5B was identified in about 1–2% of patients with non-small-cell lung cancer (NSCLC), who were negative for mutations or rearrangements in other common oncogenic drivers such as EGFR, HER, ERBB2, BRAF, KRAS, ALK, etc. [110,111,112]. In addition, in some patients with lung cancer M918T (MEN2B) RET mutation and fusion with other proteins was identified [102,103]. Also approximately 3% of melanocytic neoplasms are positive for RET fusion [113].

RET/PTC isoform expression was detected in breast cancer tumors where it correlated with estrogen receptor (EsR). In breast cancer cell lines, RET/PTC was expressed mostly in EsR-positive cell lines. However, RET/PTC expression was not detected in most of the EsR-negative cell lines. Estrogens were shown to transcriptionally upregulate RET/PTC expression [114].

It is important to note here that the signaling elicited by mutated RET is different in nature compared to the signaling produced by RET ligands such as GFLs. Mutated isoforms of RET are constitutively active for a long period of time. The signaling elicited by GFLs is pulsatile and self-limiting via degradation of the ligand and receptor by proteases, activation of silencing mechanisms, e.g., triggering the activation of phosphatases dephosphorylating RET [115] and negative feedback loops in intracellular signaling cascades [116,117,118]. The combination of these events leads to rapid quenching of the signal elicited by GFL. Interestingly, in the presence of the constitutively active forms of RET, the mechanisms of its negative regulation are also activated [115]. However, in this case, the persistent presence of receptor stimulation leads to oscillatory patterns of intracellular signaling (e.g., ERK signaling cascade) activation (Sidorova et al., unpublished observation), which is also predicted to occur in the presence of natural ligand. Nevertheless, in the presence of natural ligands, these oscillations are difficult to detect experimentally due to their small amplitude, short duration, and rapid changes [115]. Importantly, a constitutively active RET signals not only on the cell surface but also in ER during the process of protein maturation. RET MEN forms are already active in ER and signal on their way to the cell surface. RET/PTC variants signal in various cellular compartments [105]. Wild-type RET signaling occurs in lipid rafts where it is recruited by GFRɑ co-receptors. Transition to rafts is necessary for the efficient activation of intracellular signaling pathways and subsequent events on the cell and tissue level, e.g., cell survival, organ formation [28,119]. Mutated RET variants can also trigger intracellular cascades being outside lipid rafts, since they signals in the absence of co-receptor and the process of their recruitment to raft is GFRɑ -dependent; therefore, the pattern of activated secondary messengers can be different for wild-type and mutated RET. Despite mechanistic spatio-temporal differences in signaling, ligand-activated RET is considered to be able to contribute to the invasion of tumor cells and the progression of oncogenesis, as described in the next chapter.

#### 3.6.2. Oncogenic Potential of Wild-Type RET

Extensive in vitro data unequivocally demonstrate that in breast cancer cell lines, GDNF promotes cell migration and survival in an RET-dependent manner, rendering cells insensitive to anticancer drugs targeting EsR or aromatase. Similarly, the proliferation and survival of pancreatic and prostate cancer cell lines that often express GFRɑ1 and RET can be promoted by GDNF [120,121,122,123].

The pharmacological inhibition of RET with panspecific kinase inhibitors restores the sensitivity of breast cancer cell lines to tamoxifen, fulvestrant, and letrozole [124,125,126]. In animal models, additive effects of treatment with a combination of anti-EsR agent and RET inhibitor on tumor size were not detected, although the metastatic index in lungs was lower in the case of dual inhibition of RET with panspecific kinase inhibitors and EsR with tamoxifen [126]. There is also a link between inflammation, which often accompanies oncogenesis, and RET expression. The effects of inflammatory cytokine interleukin 6 (IL-6) on the migration of breast cancer cell lines were abolished in the presence of kinase inhibitors, although this interleukin does not activate RET directly [126]. At least in breast cancer cells, inflammatory mediators may upregulate GDNF expression, thus indirectly triggering RET signaling [125]. However, specific RET inhibitors are not available, and existing molecules target broad spectrum of various kinases, although with different affinity. IL-6 signals via glycoprotein 130, which activates multiple intracellular signaling cascades that are heavily dependent on the processes of protein phosphorylation [127]. Therefore, the treatment of the cells with a kinase inhibitor can abolish IL-6 signaling independently of RET as well.

Analysis of clinical samples collected from patients with breast tumors also demonstrates the overexpression of RET in a significant portion of these specimens. However, there is a discrepancy in the percentage of the breast tumors overexpressing RET between the data collected using immunohistochemical and mRNA-level assessment methods. Gatteli et al. reported the presence of RET protein overexpression in 74% of breast tumors and found a positive correlation between the level of RET protein and metastasis-free and overall survival. At the same time, an elevated mRNA level of RET was detected only in 30–40% of breast cancer biopsies, and this parameter did not correlate with lymph nodes metastasis or lymphovascular invasion [4,128]. On the contrary, elevated levels of GFRɑ1 mRNA were detected in almost 60% of patients’ samples, and they correlated with the invasion and metastasis of breast cancer cells. Only 18.1% of tumors were double positive for RET/GFRɑ1 based on mRNA analysis data [4]. However, RET can also transmit a signal from GDNF in a complex with soluble GFRɑ1 [9] produced by e.g., neuronal cells. The percentage of GFRɑ1-negative RET positive breast tumors in the study by Essiger et al. was 0.9%; thus, the other three GFR co-receptors are unlikely to have a major contribution in GFL-mediated effects in breast cancer [4].

While the discrepancy between immunohistochemistry and mRNA level data can be explained by the difference in the patient populations and the data analysis setup, it is also possible that technical artifacts related to the unspecific binding of RET antibodies to breast biopsies led to the overestimation of GDNF/GFRɑ1/RET role in the breast cancer. Many antibodies against GDNF, GFRɑ1, and RET are not specific and produce staining also in tissue sections from knockout animals [129]. The specific antibodies to RET have only been characterized in rodents a few years ago [113]. Therefore, it is important to support immunohistochemical findings with the data on the transcription of these genes.

The overexpression of RET was also detected by immunohistochemical methods in 40–65% of samples from pancreatic tumors and 20–75% of samples from prostate cancer as well as in samples from other cancers (reviewed in detail by Mullican, 2019 [24]), and it is generally correlated with worse prognosis and more advanced tumor stages [102,121,122]. There are also immunohistochemical data showing the overexpression of GFRɑ1 and co-expression of GFRɑ1 and RET in these specimens, at least in some cases. However, similar to breast cancer data, no significant correlation between the expression of other GFL co-receptors and prognosis for pancreatic cancer patients was identified. Taking into account the data for breast cancer samples described above, it is obvious that a more detailed characterization of biopsies from patients with pancreatic, prostate, and other cancers for the expression of components of GFL signaling complex using more reliable methods of mRNA level analysis can actually change the overall impression regarding the role of RET in these malignancies.

RET differs from other receptor tyrosine kinases in regard to kinase domain activation by phosphorylation. RET has intrinsic catalytic activity, and its enzymatic activity is only slightly increased upon the phosphorylation of tyrosine residues, at least in in vitro settings [5]. This can imply the presence of inhibitory mechanisms in the cells limiting the intrinsic activity of RET. These mechanisms can be overloaded in the case of RET overexpression, and RET can become activated in the absence of ligand.

Evidence collected in cell cultures showing the effects of pharmacological RET inhibition on survival and proliferation should be interpreted with caution. Specific or even highly selective RET inhibitors are yet to be developed. Compounds used in such research target multiple intracellular kinases. Considering the central role of phosphorylation processes for cell functioning and division, it is not surprising that in the presence of panspecific kinase inhibitors, the survival of cancer cells is diminished. With this, we would like to stress that we by no means try to belittle the relevance of these kinase inhibitors in cancer therapy. However, the data produced with these inhibitors in cancer cell lines shed little light on the role of RET in oncogenesis.

It is important to remember that GDNF in complex with GFRɑ1 can also signal RET independently via neural cell adhesion molecule (NCAM) and GFRɑ1 independently through syndecan-3 [130,131]. Generally, GFRɑ1 is expressed in the organism more widely than RET. Since, based on an analysis of mRNA levels, the overexpression of GFRɑ1, but not RET, in the breast cancer has been shown to be associated with cancer metastasis and invasion [4], it is possible that RET-independent, GDNF, and GFRɑ1-dependent events play a significant role in the tumor malignization process. Further studies are needed to clarify the role of each component in the GDNF/GFRɑ1/RET pathway in regard to its oncogenic potential.

Recent evidence obtained in mice overexpressing GDNF in moderate levels (by 2 fold compared to wild-type littermates) revealed no enhancement of tumor formation during their life span [132]. In addition, infusions of GDNF protein or the overexpression of NRTN from viral vectors in the brain of PD patients as well as systemic injections of ARTN to neuropathic pain patients did not seem to be associated with oncogenesis [98]. Thus, the ligand-induced pulsatile activation of wild type non-overexpressed RET by natural or artificial ligands may not be related with tumor formation or progress and thus can be safe for patients.

## 4. Targeting RET with Small Molecule for the Treatment of Diseases

RET plays an important role in the maintenance and survival of both dopamine and sensory neurons, as well as retinal cells. In addition, activating RET in the brainstem region can be a therapeutic strategy for the treatment of obesity. GFLs are considered as potential therapeutics agents for the treatment of various diseases. However, they are not drug-like molecules. GFLs do not cross the blood–brain barrier (BBB); therefore, they have to be delivered via complicated brain surgery in PD. GFLs have higher affinity to the extracellular matrix and proteoglycans, which results in poor distribution in the tissues. In addition, production, stability and long-term storage are the other challenges of protein drugs. Protein drugs can very easily be susceptible to both physical and chemical damages, often making them biologically inactive. Further, recombinant protein drugs have a shorter half life, making them unsuitable for therapy [133].

GFLs often have more than one target receptor. For example, GDNF functions via heparan sulfate proteoglycan syndecan-3 [134], NCAM [91,92,93], and RET receptor. This might result in undesirable effects of GFLs. Therefore, developing small molecules targeting RET selectively would solve the drawbacks associated with GFLs as drugs. Small molecules can cross BBB and may have better tissue distribution then GFLs. In addition, small molecules can be given orally or through injection, by which complicated surgery needed for their delivery to the brain can be avoided.

We have screened and developed the first and second generation of three structurally unrelated RET agonists (BT, HUS, and Q compounds) and tested them both in in vitro and in vivo assays [3,44,45,74,135,136]. Compounds from the BT scaffold were tested in animal models of PD and neuropathic pain. BT13 was shown to support the survival of naive cultured dopamine neurons, protect cultured dopamine neurons from toxin-induced cell death, and promote neurite outgrowth from cultured sensory neurons [3]. BT13 was also able to alleviate motor deficits in the 6-OHDA model of PD as well as attenuate neuropathy-induced pain-like behavior in the rat neuropathic pain model [135]. The second generation BT compounds, BT44, alleviated pain in surgery-based and diabetes-induced models of NP [136]. The second and the third group of RET agonists (Q and HUS compounds), which have better pharmacokinetic and pharmacodynamics properties than BT compounds, support the survival of photoreceptor neurons in ex vivo animal model of RP and activate prosurvival intracellular signaling in retina in vivo [3,74]. However, Q and HUS compounds have not been tested in other disease models such as PD, neuropathic pain, and the animal model of obesity. Further development of these compounds can eventually result in the generation of disease-modifying drugs against neurodegeneration and obesity.

The potential mechanism of action on the molecular level was studied for BT compounds using molecular dynamic simulation and docking methods. The combination of in silico and in vitro data indicates that these small molecules most likely bind to RET on RET/GFRɑ interaction interface, thus mimicking a complex of GFL-GFRɑ [137]. This possibly results in a change in RET conformation and increase in RET kinase activity, resulting in RET phosphorylation and the subsequent activation of intracellular cascades. However, further studies are needed to understand if other agonists target the same binding site and to identify molecular changes occurring in the RET molecules after the stimulation with small molecular weight agonist.

Due to the well-established role of RET in various types of cancers, its antagonists are important for antitumor therapy. The development of specific RET inhibitors is rather challenging, since the RET kinase domain is similar to that of other RTKs. However, a number of small molecular weight kinase inhibitors approved for anti-cancer therapy also act as RET antagonists. These molecules also target other RTKs, among those vascular endothelial growth factor receptors. Although this feature makes it difficult to dissect the effect of only RET inhibition in cancer treatment, it can be very useful from the therapeutic point of view, because such compounds can also reduce tumor vascularization. In addition, the process of tumor evolution may lead to the development of resistance to the compounds specifically targeting a single RTK. Thus, the identification of specific RET antagonists is an interesting scientific task, and it is important to understand the role and mechanism of RET involvement in carcinogenesis, but therapeutically, such inhibitors may be less attractive. Therefore, current efforts in this field are mainly focused on the development of polyspecific kinase inhibitors with acceptable safety profiles. A detailed review describing the effects, targets, and specificity of individual kinase inhibitors with a focus on RET was recently published by Falco and co-authors [79,138].

The limitation of the present review is in it’s scope. Here, we mostly focused on various conditions where both RET and GFLs are extensively studied, and the clinical potential of RET modulation is well-established. Therefore, this review is limited to the potential role of RET in some neurodegenerative diseases, cancer, and obesity. However, RET is expressed in several various tissues and also in different neuronal populations such as dopamine neurons, motor neurons, sympathetic neurons, and parasympathetic neurons [31,32,33,139]. RET also regulates development of the kidney [39], but importance of RET in kidney diseases has not been reported yet. Further, RET-dependent signaling may play a role in other diseases and conditions e.g., amyotrophic lateral sclerosis (ALS) [140] and addiction [141]. In some tissues e.g., the hippocampus, the expression of RET is negligible in normal conditions but can be upregulated upon lesion [142]. RET can be also differently expressed in tissues of experimental animals and humans. Therefore, the modulators of RET signaling can on one hand be evaluated for efficacy in a number of different conditions, but on the other hand, the developed agonists may produce some target-related adverse effects. Therefore, further studies are needed to evaluate the role of each component of GDNF/GFRɑ/RET in health and disease states and develop efficient therapeutics targeting these proteins.

## 5. Conclusions and Perspectives

Due to the importance of RET-dependent signaling for neuronal survival and appetite control, targeting this pathway with agonists may result in the development of novel disease-modifying treatments against neurodegenerative disorders, chronic pain, and obesity, all of which represent major challenges for healthcare in the modern world. Attempts to use natural ligands of RET, which are GFL proteins for this purpose, achieved so far rather limited success because of their poor pharmacological characteristics. Obvious alternatives to GFLs for clinical use are small molecular weight agonists of RET, GFRɑ, or GFRɑ/RET as well as GFL-derived peptides, a few of which have recently been discovered. However, the development of these compounds has been hindered by concerns regarding the oncogenic potential of RET activation. Based on the data presented in the current review, it is clear that understanding the role of wild-type RET in oncogenesis requires further studies. Available data suggest that GFRɑ rather than RET may be involved in the malignization process, while the short-term pulsatile moderate activation of wild-type non-overexpressed RET by natural or artificial ligands can be safe for patients. Therefore, RET agonists targeting RET described in the review can represent an important step forward in the development of novel treatments for neurodegeneration, pain, and obesity.

## Abbreviations

AAv	Adeno-associated virus
ARTN	Artemin
BBB	Blood–brain barrier
CERE-120	Adeno-associated virus-encoded neurturin
ER	Endoplasmic reticulum
ERK	Extracellular signal-regulated kinases
EsR	Estrogen receptor
GFLs	GDNF family ligands
GDNF	Glial cell line-derived neurotrophic factors
GFRAL	GDNF family receptor alpha-like
GFRα	GDNF family receptor alpha
GDF15	Growth differentiation factor-15
HSCR	Hirschsprung
MAPK	Mitogen-activated protein kinases
MEN	Multiple endocrine neoplasia
NRTN	Neurturin
NP	Neuropathic pain
PSPN	Persephin
PTC	Papillary thyroid carcinoma
PD	Parkinson’s disease
RETRP	Rearranged in transfectionRetinitis Pigmentosa
SNpc	Substantia nigra pars compacta
